# Mitochondrial biogenesis and metabolic hyperactivation limits the application of MTT assay in the estimation of radiation induced growth inhibition

**DOI:** 10.1038/s41598-018-19930-w

**Published:** 2018-01-24

**Authors:** Yogesh Rai, Richa Pathak, Neeraj Kumari, Dhananjay Kumar Sah, Sanjay Pandey, Namita Kalra, Ravi Soni, B. S. Dwarakanath, Anant Narayan Bhatt

**Affiliations:** 1 Institute of Nuclear Medicine and Allied Sciences, Delhi, 110 054 India; 2 Present Address: Shanghai Proton and Heavy Ion Center, Shanghai, China

## Abstract

Metabolic viability based high throughput assays like MTT and MTS are widely used in assessing the cell viability. However, alteration in both mitochondrial content and metabolism can influence the metabolic viability of cells and radiation is a potential mitochondrial biogenesis inducer. Therefore, we tested if MTT assay is a true measure of radiation induced cell death in widely used cell lines. Radiation induced cellular growth inhibition was performed by enumerating cell numbers and metabolic viability using MTT assay at 24 and 48 hours (hrs) after exposure. The extent of radiation induced reduction in cell number was found to be larger than the decrease in MTT reduction in all the cell lines tested. We demonstrated that radiation induces PGC-1α and TFAM to stimulate mitochondrial biogenesis leading to increased levels of SDH-A and enhanced metabolic viability. Radiation induced disturbance in calcium (Ca^2+^) homeostasis also plays a crucial role by making the mitochondria hyperactive. These findings suggest that radiation induces mitochondrial biogenesis and hyperactivation leading to increased metabolic viability and MTT reduction. Therefore, conclusions drawn on radiation induced growth inhibition based on metabolic viability assays are likely to be erroneous as it may not correlate with growth inhibition and/or loss of clonogenic survival.

## Introduction

The search of an effective radio-protector for protection of normal tissue toxicity during radio-therapy and nuclear accidents; and a newer, more potent radio-sensitizer to achieve enhanced therapeutic gain in radio resistant tumors, are the primary goals of radiation oncologists and radiation biologist. Further, the identification of promising molecule(s) from the library to develop as a radio-protector or adjuvant (radio-sensitizers/chemosensitizers) for established radio-/chemotherapy, the high throughput screening of large number of molecules are essentially required. These methods are required to give results with accuracy while handling large number of samples to develop the confidence in the process of screening.

Metabolic viability based assays using tetrazolium salts like MTT (3-(4,5-Dimethylthiazol 2-yl)-2,5-diphenyltetrazolium bromide) and MTS (3-(4,5-dimethylthiazol-2-yl)-5-(3-carboxymethoxyphenyl)-2-(4-sulfophenyl)-2H-tetrazolium) are the most commonly used method for high throughput screening of anti-proliferative property of compounds on cultured cells^1^. The tetrazolium salts used in these assays measure the mitochondrial metabolic rate and indirectly reflect the viable cell numbers^2–5^. The tetrazolium salt MTT is reduced to water insoluble purple formazan crystal in the metabolically active cells by mitochondrial dehydrogenases^6^, predominantly succinate dehydrogenase^7–10^ which can be further measured on spectrophotometers upon solubilisation. The total amount of formazan produced upon MTT reduction is directly proportional to the number of viable cells in the culture. Therefore MTT assay has been widely applied and become a standard method to evaluate cell viability^9–13^. Because only living cells having an intact mitochondria and cell membrane can catalyze the reaction; this method is used to measure the remaining viable cells after the treatment induced cell kill. Because of the low cost and ease of performing, these assays are used worldwide for analysing metabolic viability and cell proliferation^14–17^.

While studying the radiation sensitivity in various cell lines, we observed noticeable cell kill/growth inhibition at 24 and 48 hrs after radiation exposure, when cell density was observed directly under microscope or counted with a neubauer chamber; however the MTT assay results showed very minimal change in the formazan formation between control and irradiated groups. Similar observations were reported in the literature earlier also, while studying the growth inhibitory effects of polyphenols^1,18,19^. It appears, metabolic viability based assays do not give the real picture of cell viability or proliferation when compared with the actual cell numbers, in case of polyphenols^1,18,19^ and radiation (this study). These observations driven us to understand the limitation of MTT assay in the precise analysis of radiation induced growth inhibition. The limitation of MTT assay was reported earlier also^1,2,8–10^ however, it is not convincingly understood that why this assay is not able to correlate with the cell number in treated samples.

In present study, we uncover the mechanistic aspects of limitation in MTT colorimetric assay in respect to direct cell counting. Study was undertaken in widely used cell lines like NIH/3T3, Raw264.7, HEK-293, Hela, A549, MCF-7 and MDA-MB-231 which are used to study the radio-protective, radio-sensitizing and anti-cancer drug potential of various compounds with or without radiation. Our study shows that radiation induced mitochondrial biogenesis and hyperactivation of mitochondria, leading to more dehydrogenase activity per cell in treated groups resulting in enhanced substrate (tetrazolium) to product (formazan) conversion and false estimation of remaining viable cells. Our study highlights the limitation of MTT assay with mechanistic evidences of mitochondrial biogenesis.

## Results

### Changes in radiation induced metabolic viability do not correlate with growth inhibition

While studying the radiation induced growth inhibition in various cell lines using MTT assay and counting cell number, we found that results obtained from metabolic viability based assays do not correlate with actual cell number at different time points after radiation exposure. Since, the MTT assay is widely used based on the fact that it truly represents the viable cell number in any given sample^2–4^. We examined this assay by comparing the MTT values with cell number. Exponentially growing cells were exposed to ionizing radiation to analyze growth inhibition as well as metabolic viability by enumerating cell numbers and reduction of the tetrzolium salt to formazan (classical MTT assay; used here as MTT index), respectively. Primarily, cells were exposed to different doses of ionizing radiation (2, 3, 5 and 7 Gy) to observe the radiation dose dependent changes and correlation between cell number and metabolic viability. The amount of formazan formed (i.e MTT index) at 48 hrs after irradiation in all three cell lines (A549, MDA-MB-231 and HeLa) showed 20 to 35% reduction (at 5 Gy and 7 Gy; Supplementary Fig. 1Ai to Ci) as compared to un-irradiated cells, while the decrease in cell numbers was in the range of 70 to 90% (Supplementary Fig. 1Aii to Cii), clearly indicating a lack of correlation between metabolic viability (MTT index) and growth inhibition (cell numbers) at all the radiation doses; the two radiation response parameters analyzed.

**Figure 1:**
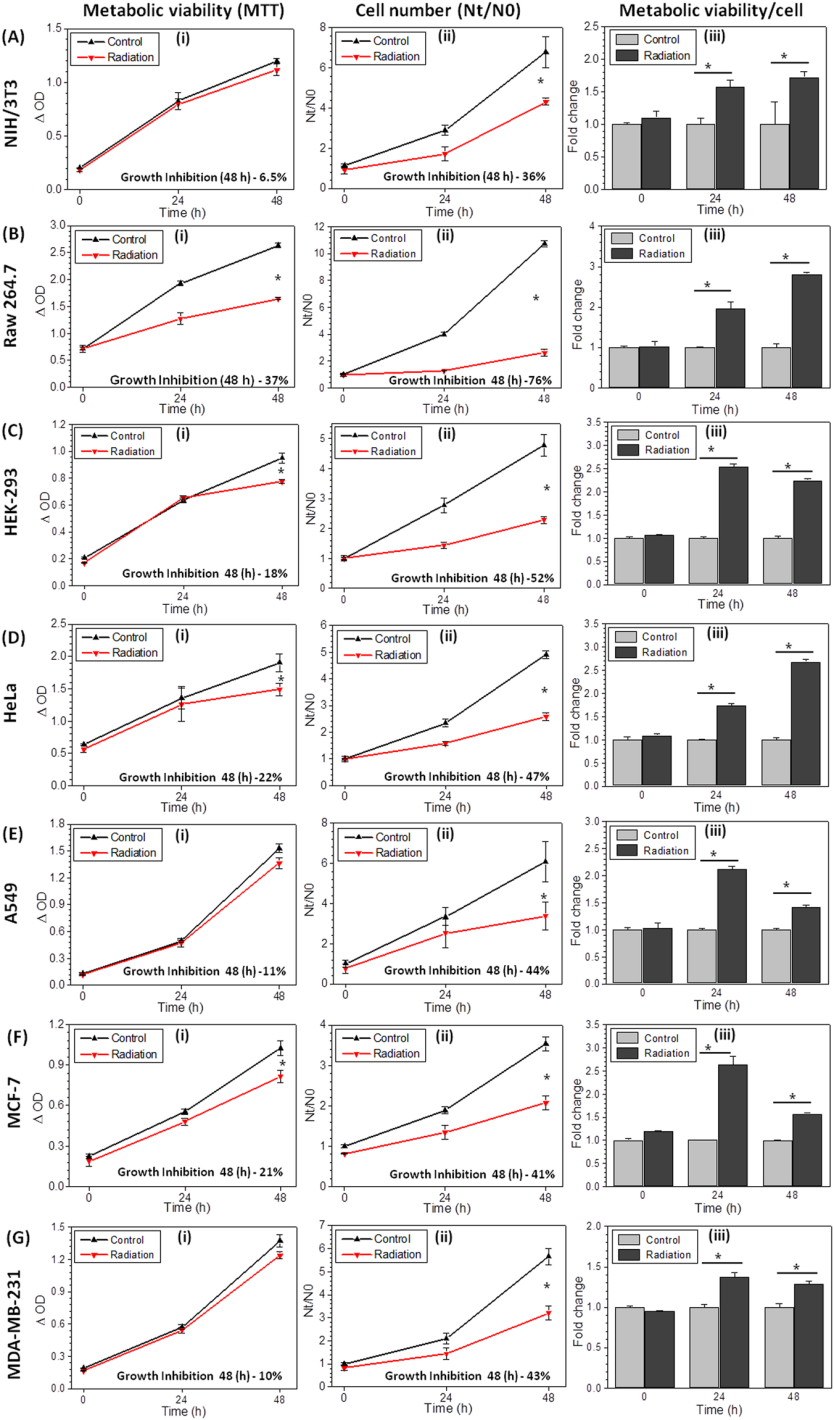
Underestimation of radiation induced growth inhibition by MTT assay. NIH/3T3 (**Ai**–**Aiii**), Raw264.7 (**Bi**–**Biii**), HEK-293(**Ci**–**Ciii**), HeLa (**Di**–**Diii**), A549 (**Ei**–**Eiii**), MCF-7(**Fi**–**Fiii**) and MDAMB-231(**Gi**–**Giii**) were analyzed using MTT assay (**Ai**–**Gi**) and cell number estimation (N_t_/N_0_, **Aii**–**Gii**) at 5 Gy radiation dose. Further MTT values (ΔOD) were normalized with its respective cell number to quantify the metabolic viability/cell (**Aiii** to **Giii**, derived information) and presented as fold change with respect to control at different time intervals. Radiation induced growth inhibition (% value) for both MTT and cell number quantified and mentioned at 48 hours (Graph inset). Star shows the statistical significance of change between the groups. Data are expressed as mean ± SD (n = 4) *p < 0.05 vs. unirradiated cells.

Further, to examine the generality of this observation, we examined the relationship between changes in cell numbers (growth inhibition) and MTT index (metabolic viability) in 7 non-tumorogenic and tumorogenic cell lines (NIH/3T3, Raw 264.7, HEK-293, HeLa, A549, MCF-7 and MDA-MB-231) at a single dose (5 Gy), which induced nearly 50% growth inhibition in the three cells tested (Supplementary Fig. 1). At 24 hrs after irradiation, all the cell lines evaluated showed either equals to control or an increase in the value of MTT index (Fig. 1Ai to Gi), except Raw 264.7 (Fig. 1Bi), which is relatively radiation sensitive. On the other hand, a significant decrease in the cell number was noted under these conditions (Fig. 1Aii to Gii). At 48 hrs following irradiation, the cell numbers were significantly lower, ranging from 43% (MDA-MB-231, Fig. 1Gii) to 76% (Raw 264.7, Fig. 1Bii) as compared to control, while MTT index values showed only 10% to 37% reduction respectively, with the maximum in Raw264.7 cell line (Fig. 1Bi). When the values of MTT index (Δ OD) were normalized with the corresponding cell number, it showed 1.4 to 3 fold (in different cell lines) enhanced metabolic viability per cell (derived information) at 24 and 48 hrs, which further reduced at 48 hrs in most of the cell lines except Raw264.7, NIH/3T3 and HeLa cells but remains significantly higher than respective control (Fig. 1Aiii to Giii). These observations clearly demonstrate that the cell number, which is the true measure of radiation induced growth inhibition and/or cyto-toxicity does not correlate with the high throughput metabolic viability based (MTT) assay, widely used for the quick assessment of radiation response, which appears to be due to the radiation induced enhanced metabolic viability (Fig. 1Aiii to Giii) of exposed cells.

### Radiation exposure augments metabolic viability by enhancing mitochondrial mass

**Figure 2:**
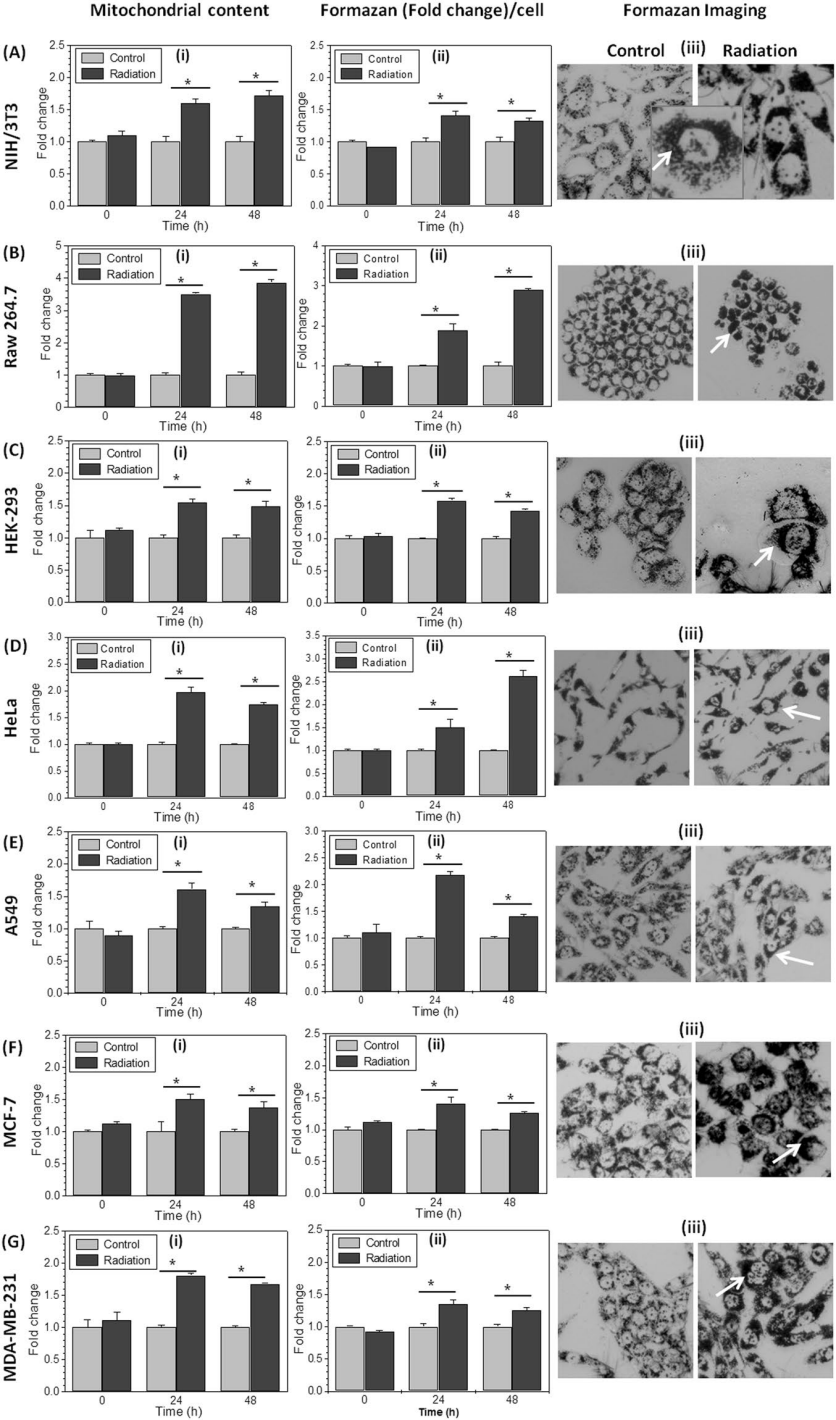
Radiation increases mitochondrial mass per cell and enhances formazan formation: The mitochondrial mass was analyzed by staining cells with MitoTracker Green FM (100 nM; 20 min) at indicated time points. Graphs **Ai- Gi** (cell lines as described in Fig. 1) showing mean fluorescence intensity (MFI), presented as fold change with respect to control. (**Aii–Gii**) The Formazan formed per cell was quantified spectro-photometrically and presented as fold change of control at respective time points in different cell lines. (**Aiii–Giii**) Photomicrograph showing formazan accumulation (at 20X objective), in control and irradiated cells captured after 2 hrs of MTT incubation at 24 hrs post irradiation in different cell lines. Zoom image of one irradiated cell is highlighted to show the enhanced punctuated formazan deposition. Data are presented as mean ± SD (n = 4) *p < 0.05 vs. untreated control cells.

The increased metabolic viability in radiation exposed cells can result from either hyperactive mitochondria or increased mitochondrial mass because conversion of MTT to formazan occurs mainly at mitochondria^7–10^. Ionizing radiation is known to induce mitochondrial mass and function in exposed cells^20,21^. Therefore, we examined if increased mitochondrial mass is responsible for enhanced MTT index in radiation exposed cells using flow cytometry. In line with earlier observations we found that the average mitochondrial mass per cell was significantly increased by nearly 1.4 fold in MCF-7 (minimum, Fig. 2Fi) to 4 fold in Raw 264.7 (maximum, Fig. 2Bi) at 24 and 48 hrs after radiation exposure (Fig. 2Ai to Gi). Simultaneously in irradiated cells enhanced production of formazan per cell also observed at respective time points, quantified under similar experimental conditions (Fig. 2Aii to Gii). The microscopic images at 24 hrs after radiation exposure also show visibly enhanced formazan deposition in irradiated cells with respect to their control (Fig. 2Aiii to Giii). The formazan containing bodies (mitochondria) in control cells are less intense in color, sparsely and uniformly distributed in the cytoplasm, whereas in the radiation exposed cells they were dark in color and clustered in perinuclear region (Fig. 2Aiii to Giii). These microscopic pictures provide visual and supportive evidence for the higher MTT index or enhanced metabolic viability per cell in irradiated cells. Further, it validates how reduced number of irradiated surviving cells can produce much higher amount of formazan (color density) per cell than respective untreated control cells, resulting in false interpretation of data.

### Radiation enhances metabolic viability by inducing mitochondrial biogenesis

Mitochondria is the major site where MTT is reduced to Formazan^7–10^, therefore increase in mitochondrial mass can increase the metabolic viability. The increased mitochondrial mass in irradiated cells could be obtained due to two reasons first, ionizing radiation induces G2/M block^22,23^ cells arrested in G2 phase will have higher number of mitochondria^22–24^ which can reduce more MTT to formazan in irradiated cells. Second, ionizing radiation is known to induce mitochondrial biogenesis^20,21^, resulting in increased mitochondrial mass. To test whether radiation induced G2/M arrest or mitochondrial biogenesis or both are responsible for enhanced mitochondrial mass per cell in irradiated cells, we examined both the hypothesis sequentially. Since, radiation induced enhance mitochondrial mass (Fig. 2Ai to Gi) was observed in all the cell lines, only HeLa and MDA-MB-231 cells were selected randomly for understanding the mechanisms underlying radiation induced enhanced metabolic viability.

**Figure 3:**
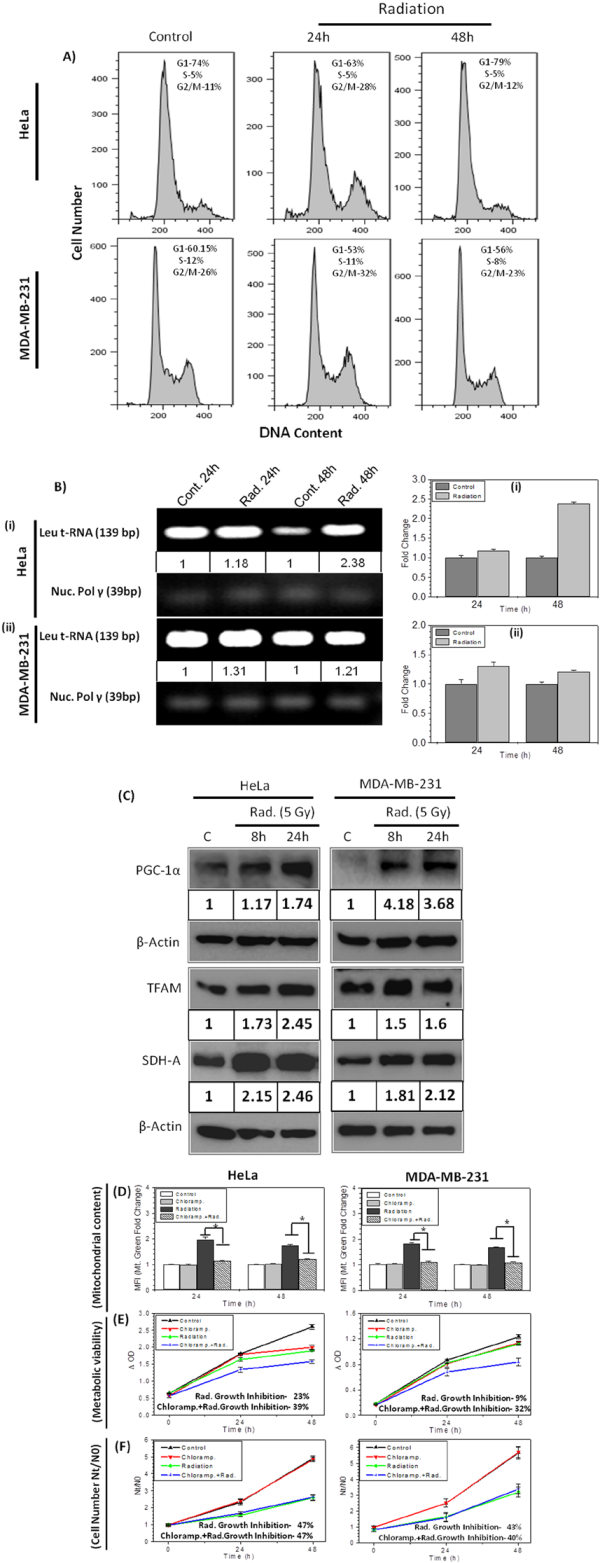
Radiation induced mitochondrial biogenesis augments metabolic viability: (**A**) Cell cycle histogram showing phase distribution (G1, S and G2/M) of cells at 24 and 48 hrs post irradiation in HeLa and MDA-MB-231 cells. (**B**) Mitochondrial genome encoded Leu tRNA gene was analyzed by semi quantitative PCR and normalized with nuclear pol gamma gene copy number. The mtDNA copy number is also presented as comparative fold change at respective time points (bar diagram). (**C**) Protein expression analysis of mitochondrial biogenesis and mitochondrial complex-II subunit SDH-A presented in HeLa and MDA-MB-231 cells. The values between the blots represent the fold increase at 8 and 24 hrs. post irradiation quantified by densitometry and normalized with respective β-Actin. The DNA (**B**) and protein blot (**C**) images were cropped from full-length blots (Supplementary Figs 2 and 3). (**D**) Analysis of effect of chloramphenicol (40 μM; 30 mins prior to IR; continuous exposure) on mitochondrial content by MitoTracker Green FM at indicated time points using flow cytometer and graphs presented as fold change of mean fluorescence intensity (MFI) with respective control. Effect of Chloramphenicol on radiation induced growth inhibition was analyzed (at 5 Gy) by MTT assay (**E**) and cell number (**F**) in HeLa and MDA-MB-231 cells. Growth inhibition quantified and mentioned at 48 hours. Data are expressed as mean ± SD from triplicates. *p < 0.05 vs. unirradiated control.

The cell cycle distribution was performed at 24 and 48 hrs after radiation. At 24 hrs, 17 and 6% excess cell populations were found in the G2/M fraction of the cell cycle in HeLa and MDA-MB-231 cells respectively. However the block is completely released at 48 hrs (Fig. 3A), suggesting that radiation induced cell cycle arrest may be partly contributing to the increased mitochondrial mass at 24 hrs but not at 48 hrs. It is also important to note that nearly 17% increased number of cells in G2/M phase cannot bring about 1.9 fold (which is 90% high) change in the mitochondrial mass and 1.5 fold change in MTT reduction to formazan at 24 hrs in HeLa cells (Fig. 2Di and Dii). Although, the cell cycle block was completely released at 48 hrs, yet the mitochondrial mass and enhanced metabolic viability remained significantly higher than the respective controls. These observations, do not support the proposition that radiation induced enhanced metabolic viability is because of cell cycle arrest mediated enhanced mitochondrial mass as reported earlier in case of polyphenolic compounds treatment^18,25^.

Further, to test the hypothesis that radiation induced hyper metabolic activity which correlates with enhanced mitochondrial mass in exposed cells (Fig. 2Ai to Gi) is because of radiation induced mitochondrial biogenesis, we analysed the mtDNA copy number in control and radiation exposed cells. The copy number of Leu t-RNA gene was measured for mitochondrial genome encoded mtDNA copy number and normalised with nuclear pol-gamma, using semi-quantitative PCR method. Both HeLa and MDA-MB-231cells showed 18% and 31% enhanced mtDNA copy number after 24 hrs of radiation exposure, which further increases up to 138% in HeLa and remains 21% high in MDA-MB-231 than control cells at 48 hrs (Fig. 3B). Further, we examined the time dependent expression level of PGC-1α (Peroxisome proliferator-activated receptor gamma coactivator 1-alpha) and TFAM (Mitochondrial Transcription Factor A) proteins using Western blot. These two proteins are the key regulator of mitochondrial biogenesis and maintenance in cells^26–28^. Interestingly, time dependent increase in both PGC-1α and TFAM expression (Fig. 3C) was observed in cells (HeLa and MDA-MB-231) exposed to radiation, which correlates with enhanced mitochondrial mass at 24 hrs time point (Fig. 2Di and Gi). Further we checked, if enhanced mitochondrial mass is functional and has enhanced expression of protein SDH-A, which is primary reducing enzyme of MTT into Formazan^7–10^. The protein levels of SDH-A was also found increased after 8 and 24 hrs of radiation exposure (Fig. 3C), which correlates with enhanced MTT index or enhanced metabolic viability in HeLa and MDA-MB-231 cells (Figs 1 and 2). SDH-A levels were found significantly increased in other cell lines also (data not shown). Further to validate the hypothesis, radiation induced mitochondrial biogenesis results in increased metabolic viability; we inhibited the mitochondrial biogenesis using chloramphenicol^29,30^. We found that chloramphenicol, at non-toxic concentration reduced radiation induced mitochondrial biogenesis significantly, in both the cell lines (Fig. 3D).When MTT and growth kinetics (Fig. 3E and F) were performed in cells treated with chloramphenicol before radiation exposure, it showed significantly low Formazan formation than radiation alone (Fig. 3E), thereby suggesting radiation induced enhanced metabolic viability is mainly due to mitochondrial biogenesis. The difference between radiation induced growth inhibition curve obtained from MTT assay and cell counting remains only 8% in chloramphenicol treated cells (Fig. 3E and F), which were 25% and 33% in HeLa and MDA-MB-231 cells (Fig. 1Di-ii and Gi-ii), respectively. These results substantiate the hypothesis that radiation induced enhance mitochondrial mass and metabolic viability comes from radiation induced mitochondrial biogenesis up to large extent, which is regulated by radiation induced PGC-1α and TFAM.

### Radiation induces hyperactivation of mitochondria

**Figure 4:**
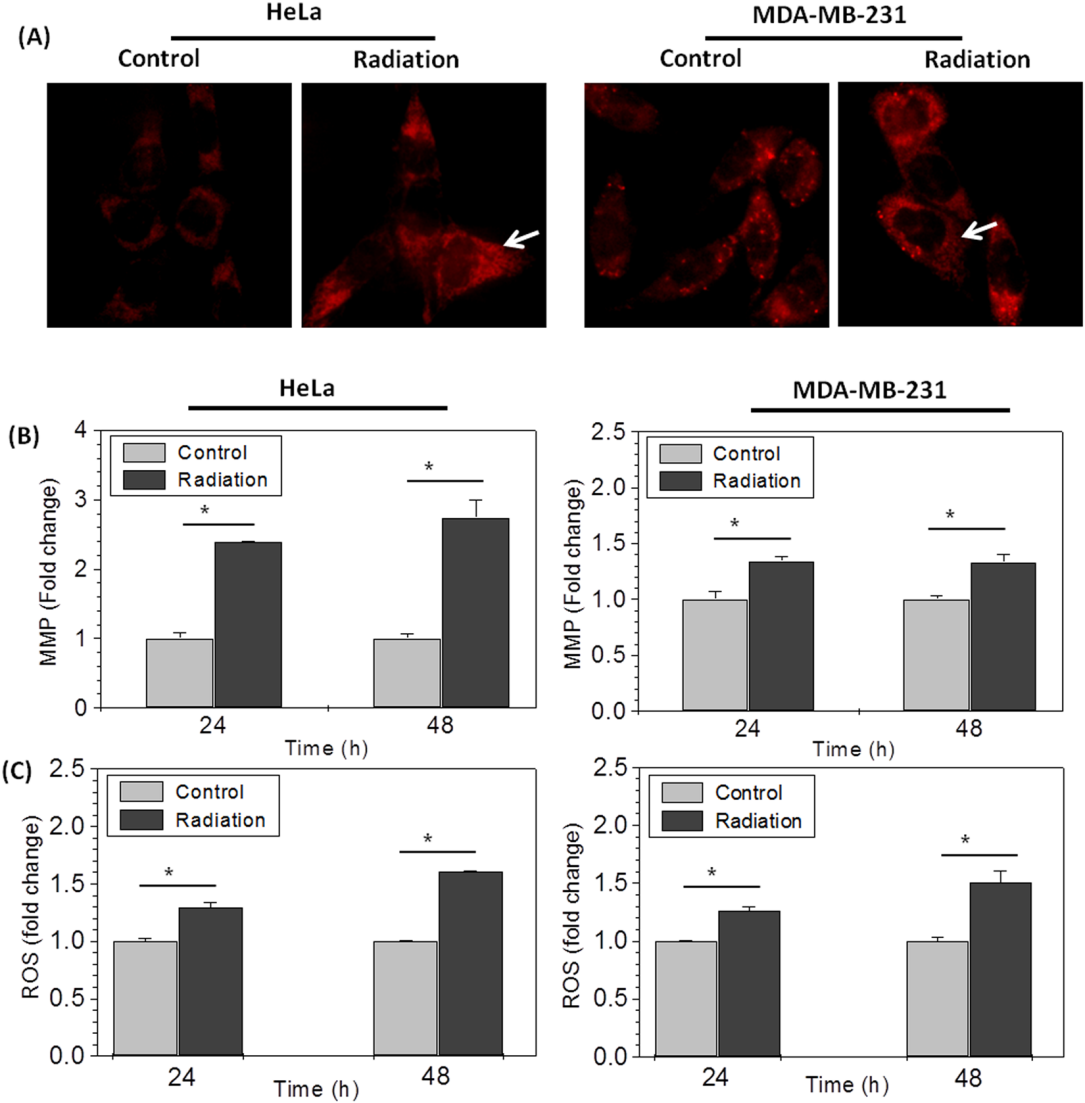
Radiation induces hyperactivation of mitochondria: (**A**) Photomicrographs showing mitochondrial membrane potential (MMP) in HeLa and MDA-MB-231 cells stained with TMRM (5 nM/ml; 30 min; 37 °C) at 24 hrs post irradiation. Radiation induced changes in mitochondrial MMP was quantified by DiOC6 **(B)** and superoxide radical was quantified using MitoSox (**C**) in HeLa and MDA-MB-231 cells using flow cytometer and presented as MFI fold change at indicated time points with respect to control. Star shows the statistical significance of change between the indicated groups. Data are expressed as mean ± SD from triplicates. *p < 0.05 vs. unirradiated control.

In earlier results, we observed that ionizing radiation induces the mitochondrial mass in cells, which seems to be responsible for enhanced metabolic viability (MTT index) in irradiated cells; however it is known that radiation also induces hyper activation of individual mitochondria in irradiated cells^31^. To determine whether enhanced MTT index was observed due to enhanced mitochondrial mass only or radiation induced hyperactivation of mitochondria also, we measured ΔΨm (mitochondrial membrane potential, MMP) by fluorescence microscopy using TMRM. Irradiated cells (at 24 hrs.) showed bright red punctated mitochondria suggestive of high ΔΨm, as compared to control (Fig. 4A). This result was further validated by quantitative estimation of ΔΨm using DiOC6 by flow cytometry. HeLa cells showed 2.4 to 2.8 fold increased MMP however, MDA-MB-231 cells showed 1.3 fold changes in radiation induced MMP at both the time points, respectively (Fig. 4B). This high mitochondrial membrane potential correlates with multifold high formazan formation in each mitochondrion of irradiated cells (inset picture in Fig. 2Aiii), suggesting hyper complex II (SDH) activity (Fig. 2Aii to Gii), probably due to radiation induced increased ΔΨm. This observation is in line with earlier findings in other studies, which suggest that Complex II is more efficient in establishing and maintaining ΔΨm under stress conditions^32,33^. The increased ΔΨm due to hyperactive complex II ensures enhanced activity of other mitochondrial dehydrogenases also, which may be contributing to increased formazan formation other than SDH.

Succinate driven oxidation via complex-II (SDH) have been found with the significant contribution towards the enhanced mitochondrial ROS generation^34^. Therefore, we analyzed the mitochondrial superoxide level in control and irradiated cells to test the fact that hyper metabolically active mitochondria should produce more superoxide radical. Cells stained with MitoSox (mitochondrial ROS indicator) were analyzed on flow cytometer. Irradiated cells show significantly enhanced MitoSox fluorescence than their respective control. Both the cell lines showed nearly 1.25 fold increase in mitochondrial ROS at 24 hrs however, it further increased to 1.65 fold in HeLa and 1.5 fold in MDA-MB-231 cells at 48 hrs after radiation exposure (Fig. 4C). Since mitochondrial dehydrogenase SDH is the main contributor of MTT reduction^7–10^, these results validate the observation of increased ROS is directly proportional to the enhanced SDH activity^34^. These results suggest that enhanced mitochondrial mass consists of functionally hyperactive mitochondria, reducing larger amount of MTT in formazan per cell in irradiated samples.

### Radiation induces Calcium accumulation in mitochondria

Ionizing radiation disturbs the cellular calcium homeostasis leading to enhanced release of calcium from endoplasmic reticulum (ER) to cytoplasm, which is then buffered by functional mitochondria in the perinuclear region of the cells^35,36^. Altered calcium homeostasis also induces ROS (reactive oxygen species) production and mitochondrial biogenesis^35–37^.

**Figure 5:**
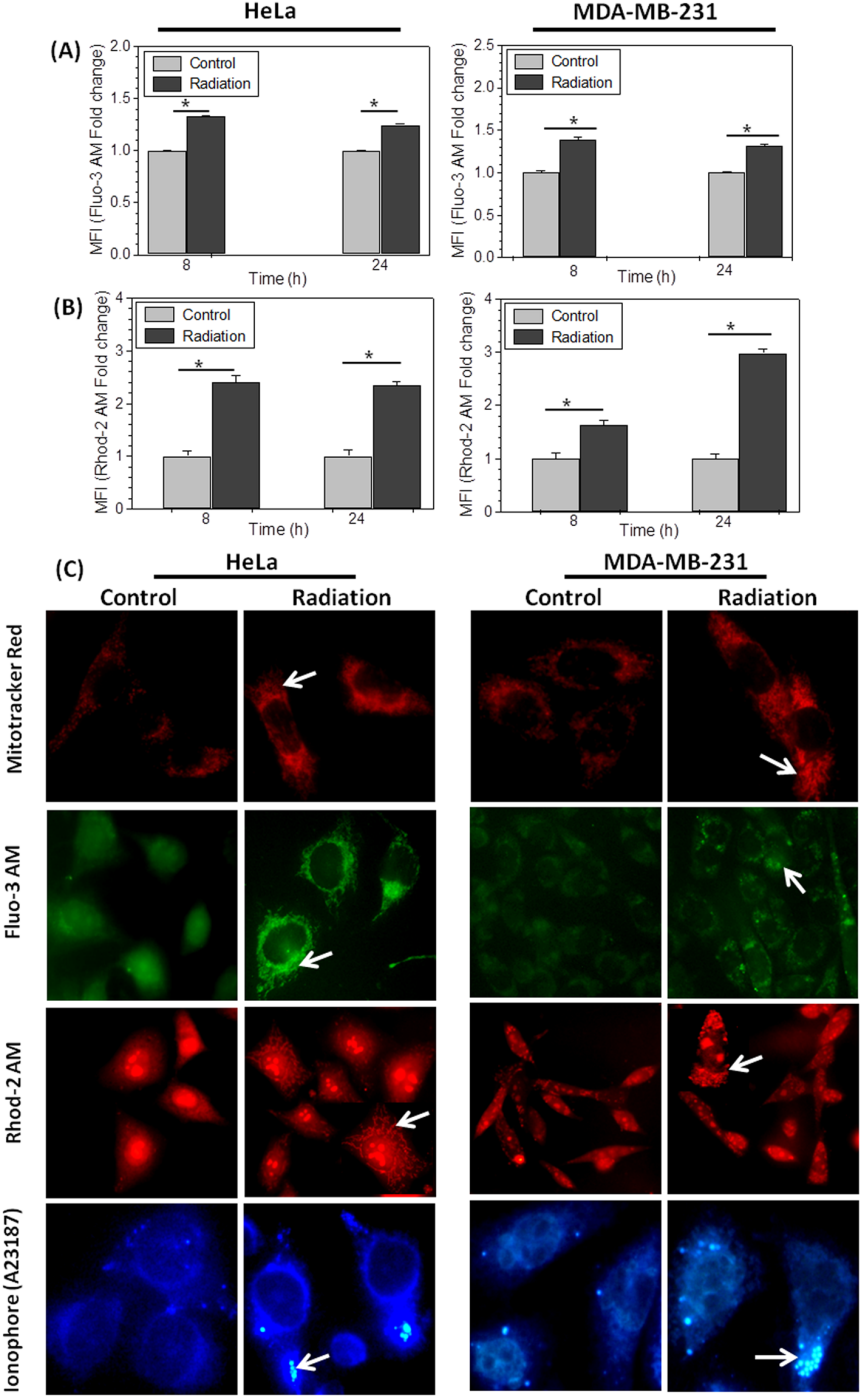
Radiation induces mitochondrial Ca^2+^ accumulation: (**A** and **B**) Bar diagram shows alteration in Ca^2+^ concentration in HeLa and MDA-MB-231 cells stained with Fluo-3 AM (5 µM; 30 min) and Rhod-2 AM (1 µM; 20 min; 37 °C), analyzed using flow cytometer. MFI fold change is presented here at 8 and 24 hrs post-irradiation with respect to control. (**C**) Photomicrograph shows the radiation induced change in mitochondria using MitoTracker® Red and cellular distribution of Ca^2+^ stained with Fluo-3AM and mitochondrial calcium using Rhod-2 AM, fluorescent probes. Zoomed images of Rhod-2 AM stained single cell is highlighted for better appreciation of radiation induced accumulation of mitochondrial Ca^2+^. Fourth row shows A23187 (6 µM, 30 mins) stained cells, showing Ca^2+^ loaded mitochondria in HeLa and MDA-MB-231cells. Images were captured under fluorescence microscope with 40X objective.

To investigate, if increased metabolic viability observed at 24 and 48 hrs is because of enhanced cytoplasmic and mitochondrial Ca^2+^ levels, free cellular and mitochondrial Ca^2+^ was estimated. The control and treated cells were stained with Fluo-3AM (Ca^2+^ indicator) and Rhod-2AM (specific mitochondrial Ca^2+^ indicator)^38^ at respective time points and analyzed on flow cytometer. The significant increase in the level of calcium was observed in irradiated cells, which was increased by nearly 1.4 fold (40%) in both the cell lines (HeLa and MDA-MB-231) investigated, as suggested by increased Fluo-3 fluorescence at 8 hrs, which reduces marginally at 24 hrs (Fig. 5A). Interestingly, even more radiation induced increase in mitochondrial Ca^2+^ (2.4 fold in HeLa and 1.6 fold in MDA-MB-231, Fig. 5B) was noted. This observation was further verified by visualizing the stained cells under fluorescence microscope. Cells stained with Mitotracker Red, Fluo-3AM and Rhod-2AM independently at 24 hrs after radiation treatment was observed. Fluo-3AM and Rhod-2AM both shows strong perinuclear punctated fluorescence with same pattern as shown by mitotracker red in radiation exposed corresponding cells (Fig. 5C), suggesting that increased cytoplasmic Ca^2+^ is accumulated in mitochondria. Moreover, specific Ca^2+^ signals from mitochondria in Rhod-2AM stained cells, validate this observation. Interestingly, MDA-MB-231 cells showed already high mitochondrial Ca^2+^ (Rhod-2-AM staining) and perinuclear mitochondrial clustering (Mitotracker Red staining) in control cells, which increases further after irradiation, this could be the reason why this cell showed lowest increase in radiation induced mitochondrial biogenesis and formazan formation, subsequently. We have demonstrated in our earlier study that radiation causes accumulation of Ca^2+^ in mitochondria leading to mitochondrial damage and mitophagy^36^. Mitochondria with high Ca^2+^ and enhanced mitochondrial membrane potential accumulate more A23187 and shows brighter fluorescence under microscope on UV excitation. However, permanently damaged mitochondria show very high fluorescence and appear as round bodies inside the cell^36^. In order to observe Ca^2+^ accumulation in mitochondria under these experimental conditions, we stained control and irradiated HeLa and MDA-MB-231 cells at 24 hrs after exposure (Fig. 5C). Irradiated cells also showed higher accumulation of bodies indicating radiation induced damaged mitochondria. Irradiated cells show higher Mitotracker Red, Fluo-3AM and Rhod-2AM fluorescence in perinuclear region (Fig. 5C), where mitochondria forms a network with endoplasmic reticulum (ER) and accumulates most of the stress induced Ca^2+^ leakage from ER and protect the cells from death^39^. This finding suggests that Ca^2+^ released from stores after radiation exposure is mainly accumulated in mitochondria. It is reported earlier that high Ca^2+^ in mitochondrial matrix augments SDH activity^40^. Therefore, we correlate that Ca^2+^ accumulated in mitochondria increased the SDH complex activity leading to hyperactive mitochondria and increased metabolic viability in radiation exposed cells.

### Calcium accumulation in mitochondria leads to cellular hyperactive metabolic state

**Figure 6:**
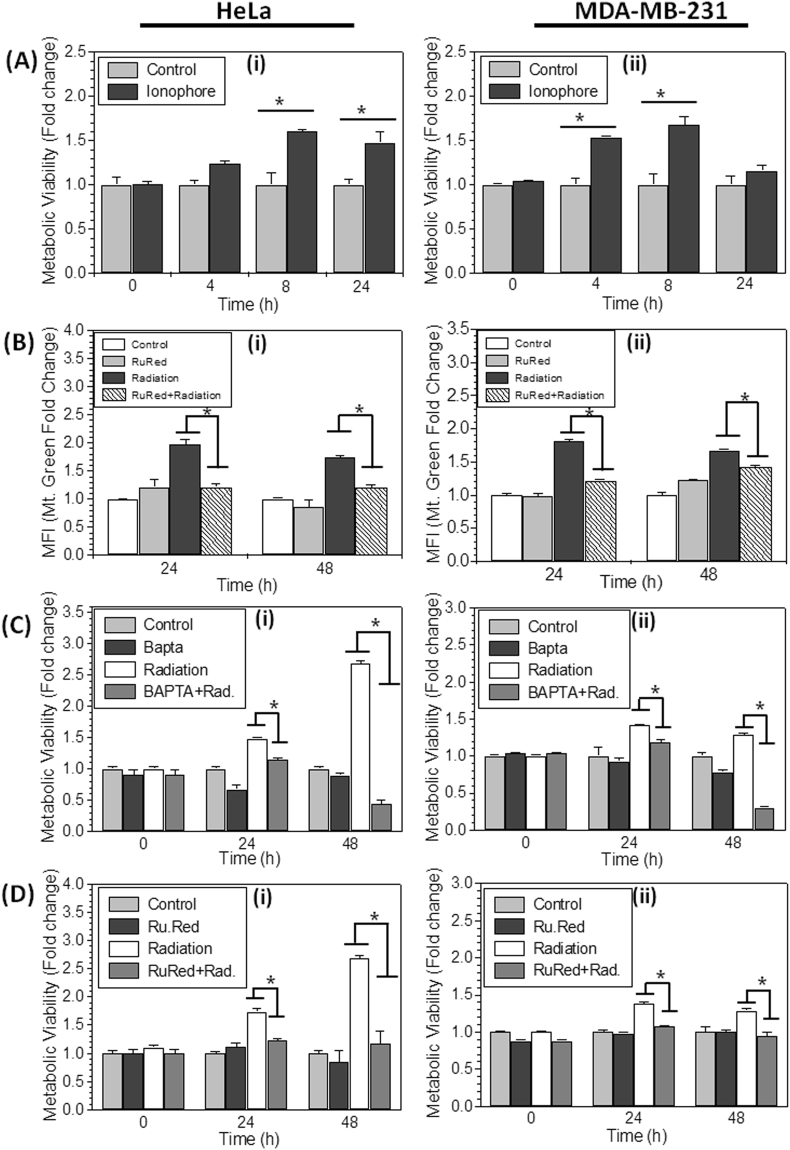
Role of Calcium in enhanced metabolic viability: (**A**) Cells were treated with A23187 (2 µM; 1 hr) to release ER stored Ca^2+^ for 4, 8 and 24 hrs and MTT index was measured by MTT assay in HeLa and MDA-MB-231 cells. The result is presented as fold change of metabolic viability/cell and normalized with respect to control. (**B**) Mitochondrial content was analyzed by flow cytometer using MitoTracker Green in Ruthenium Red (1 µM, continuous exposure) treated cells and presented as relative fold change with respect to control. (**C** and **D**) HeLa and MDA-MB-231 Cells were treated with Ca^2+^ chelator BAPTA-AM (20 µM for 2 hrs), and Ruthenium Red before irradiation and further metabolic viability was measured and presented as fold change difference with respect to control. Data are presented as mean ± SD from four independent experiments *p < 0.05 vs. control.

Further, to substantiate the role of Ca^2+^ in enhanced formazan formation, we tested if Ca^2+^ disturbance in cells alone can increase the metabolic viability. To test this proposition, cells were treated with Ca^2+^ ionophore A23187 (2 µM, which increases the cytoplasmic Ca^2+^, same as ionizing radiation) for 1 hour and analyzed the metabolic viability at 4, 8 and 24 hrs after treatment. Interestingly, cells treated with A23187 showed significantly increased (1.6 fold) metabolic viability per cell at 8 & 24 hrs in HeLa and 4 & 8 hrs in MDA-MB-231 (Fig. 6A), after treatment. These results suggest that enhanced cytoplasmic Ca^2+^ increases metabolic viability, by increasing the accumulation of Ca^2+^ in mitochondria (Fig. 5C). We also checked if, inhibition of Ca^2+^ accumulation in mitochondria can hamper the radiation induced increase in mitochondrial mass. Interestingly, we found that cells treated with inhibitor of mitochondrial Ca^2+^ uniporter, Ruthenium Red (RuR, which inhibits the accumulation of Ca^2+^ in mitochondria) showed significantly low increase in mitochondrial content (radiation induced) as compared to radiation alone (Fig. 6B). It is known that increased Ca^2+^ induces mitochondrial biogenesis through CaMKII (Calmodulin Kinase-II)^37,41^, which is also a mitochondrial resident protein known to regulate PGC-1α expression^37,41,42^. Therefore we conclude, inhibition of Ca^2+^ accumulation in mitochondria using ruthenium red reduced the mitochondrial mass, probably by inhibiting the mitochondrial biogenesis. Further, to validate if radiation induced increased cytoplasmic Ca^2+^ and its accumulation in the mitochondria leads to enhanced metabolic viability in irradiated cells, we treated the cells with BAPTA-AM (an intracellular calcium chelator) and RuR, 30 minutes before irradiation. The chelation of increased cytoplasmic Ca^2+^ using BAPTA-AM (Fig. 6C) and inhibiting the accumulation of Ca^2+^ in mitochondria using RuR (Fig. 6D), both abrogated the radiation induced increased metabolic viability after 24 and 48 hrs of irradiation in HeLa and MDA-MB-231 cells (Fig. 6C and D). These results suggest that radiation induced disturbance in Ca^2+^ homeostasis also play an important role in hyperactivation of mitochondria and enhanced metabolic viability, probably upstream to mitochondrial biogenesis.

## Discussion

MTT assay was introduced for rapid estimation of proliferation and cell viability by Mosmann because he demonstrated that MTT reduction to formazan is proportional to the number of metabolically viable cells in culture^6^. Based on his observations and its rapid nature, MTT and many more metabolic viability based assays became most popular methods in majority of the laboratories, in last several decades. However, the limitations of MTT or other metabolic viability based assays in precise estimation of proliferation and metabolic viability of polyphenols was brought to the notice and investigated, previously^1,18,19^. While studying the radio-sensitivity and resistance of several cells lines, we found that MTT assay gives underestimation of growth inhibition potential of ionizing radiation.

We validated the hypothesis that the cell proliferation data obtained from MTT assay represent underestimation of radiation induced cell kill when compared with cell number at respective time points in a number of cell lines (Fig. 1). When the amount of formazan formed (optical density) were normalized with corresponding cell numbers, irradiated cells showed multi fold increase in amount of formazan per cell (Fig. 1Aiii to Giii), which is widely interpreted as enhanced metabolic viability. This observation is substantiated by the microscopic images of formazan (After MTT reduction) control and irradiated cells. Irradiated cells show very high formazan deposition per cell than control (Fig. 2Aiii to Giii). This result clarified that radiation induces the metabolic viability in exposed cells leading to more MTT reduction per cell, which is the deviation from the principle of MTT assay that metabolic viability is directly proportional to the cell number. According to the principle of MTT or other metabolic viability based assays, more reduction of MTT means more number of viable cells, this leads to the false estimation of more number of surviving cells in radiation exposed groups and underestimation of cyto-toxic potential of ionizing radiation.

We further investigated how radiation induces metabolic viability in exposed cells? Radiation is known to induce mitochondrial mass^20,21^ which is confirmed in this study (Fig. 2Ai to Gi), suggesting that radiation induced enhanced mitochondrial mass increases the metabolic viability in exposed cells. The earlier report on limitation of MTT assay in false measurement of cyto-toxic effects of polyphenols^1^, suggest that treatment induced cell cycle arrest in G2/M phase leads to increased mitochondrial mass^22,24^ and enhanced metabolic viability^18^. Radiation also induces G2/M cell cycle arrest but nearly or more than 2 fold increase in MTT reduction (in different cell lines, Fig. 1) does not correlate with just 17% (maximum observed in HeLa) increased number of cells in G2/M phase (Fig. 3A) at 24 hrs, which further release at 48 hrs however, metabolic viability per cell was found remain high from 1.4 fold to 3 fold (in different cell lines, Fig. 1). These findings suggest that radiation induced G2/M cell cycle arrest can be a contributory factor for enhanced metabolic viability but not a major determinant. Whereas, we confirmed radiation induced mitochondrial biogenesis by estimating mtDNA copy number and radiation induced time dependent increase in the levels of PGC-1α and TFAM (Fig. 3B and C), both these proteins are central regulators of mitochondrial biogenesis^27,28^. Increased mitochondrial biogenesis also lead to the increased expression of SDH-A (Fig. 3C), which primarily converts MTT to formazan, thereby leading to augmentation of MTT reduction and enhanced MTT index per cell in radiation exposed samples. Inhibition of radiation induced mitochondrial biogenesis using chloramphenicol, reverts the effect of radiation induced enhanced formazan formation, significantly (Fig. 3E) however, it is not abrogated completely. Therefore, we investigated factors other than mitochondrial biogenesis, responsible for enhanced formazan formation in radiation exposed cells.

Radiation is known to cause disturbance in Ca^2+^ homeostasis and more Ca^2+^ accumulation in mitochondria^35,36^. The increased Ca^2+^ concentration in mitochondrial matrix also augments the functional activity of mitochondrial dehydrogenases, mainly SDH-A^40^. We showed, radiation induced increase in the intracellular Ca^2+^concentration, which mainly accumulates in perinuclear mitochondria (Fig. 5C). It is interesting to discuss here that enhanced MTT reduction was also observed in perinuclear mitochondria, predominantly (Fig. 2Aiii-Giii). The zoom microscopic image shown in inset (Fig. 2Aiii of NIH/3T3 cell) shows not only increased number of mitochondria but each mitochondrion is reducing more MTT. Therefore, based on our finding, we propose that it is not only mitochondrial biogenesis, which enhances metabolic viability by increasing the mitochondrial mass but the accumulation of Ca^2+^ in mitochondrial matrix also increases the MMP (Fig. 4A,B) and SDH activity (Fig. 3C) resulting in enhanced metabolic viability in radiation exposed cells. It is difficult to predict the contribution of individual factors in enhanced metabolic viability, however it is very clear that radiation induced mitochondrial biogenesis is the major determinant of enhanced metabolic viability in radiation exposed cells and disturbance in Ca^2+^ homeostasis plays an important role in this signaling upstream to mitochondrial biogenesis^37,41^.

Besides radiation, many anti-cancer genotoxic drugs (Etoposide, Doxorubicin and Cisplatin), which are known to induce mitochondrial biogenesis, disturbance in Ca^2+^ homeostasis and produces ROS^36,43,44^, in cells also showed enhanced metabolic viability and underestimation of drug induced cyto-toxicity measured by MTT assay (data not shown) than direct cell counting. It suggest that the limitation of MTT assay or any metabolic viability based assay in measuring the cyto-toxicity or proliferation, is not only constrained to radiation or polyphenolic compounds but extended up to many anti-cancer drugs (Etoposide, Doxorubicin and Cisplatin) and all those agents which can stimulate metabolic modification in cells by inducing mitochondrial biogenesis.

MTT or other metabolic viability based assay works on this principle that every viable cell in a given cell line will have same metabolism therefore; the quantitation of total metabolism in a given cell will be directly proportional to the viable cell number^6^. However, with significant advancement in our understanding about the stress induced cellular metabolic remodeling, in recent past and our data in this study suggest that same cells may vary in their metabolic potential when treated with radiation or different chemical agents. Therefore, the principle of metabolic viability based assay will not be applicable in measuring the cyto-toxicity or proliferation in case of those agents. Our study evidently demonstrate that radiation induces the metabolic viability of treated cells leading to increased MTT reduction from remaining less number of cells (because of radiation or drug induced cell kill), resulting in false estimation of cell number and underestimation of actual cyto-toxic potential of radiation in that particular cell line. Since, metabolic viability does not correlate with actual percent cell kill, we recommend that it should not be used for testing the cytotoxic potential and deriving the LD_50_/IC_50_ values of radiation or other agents, which induces mitochondrial biogenesis. Based on these findings, we strongly recommend being careful with data from MTT and other metabolic viability based assays when estimating growth inhibition/cytotoxicity. However, MTT assay can be used for the estimation of metabolic and redox potential of cells because the results derived from this assay represents the metabolic status of the cell and not the cell number.

**Figure 7:**
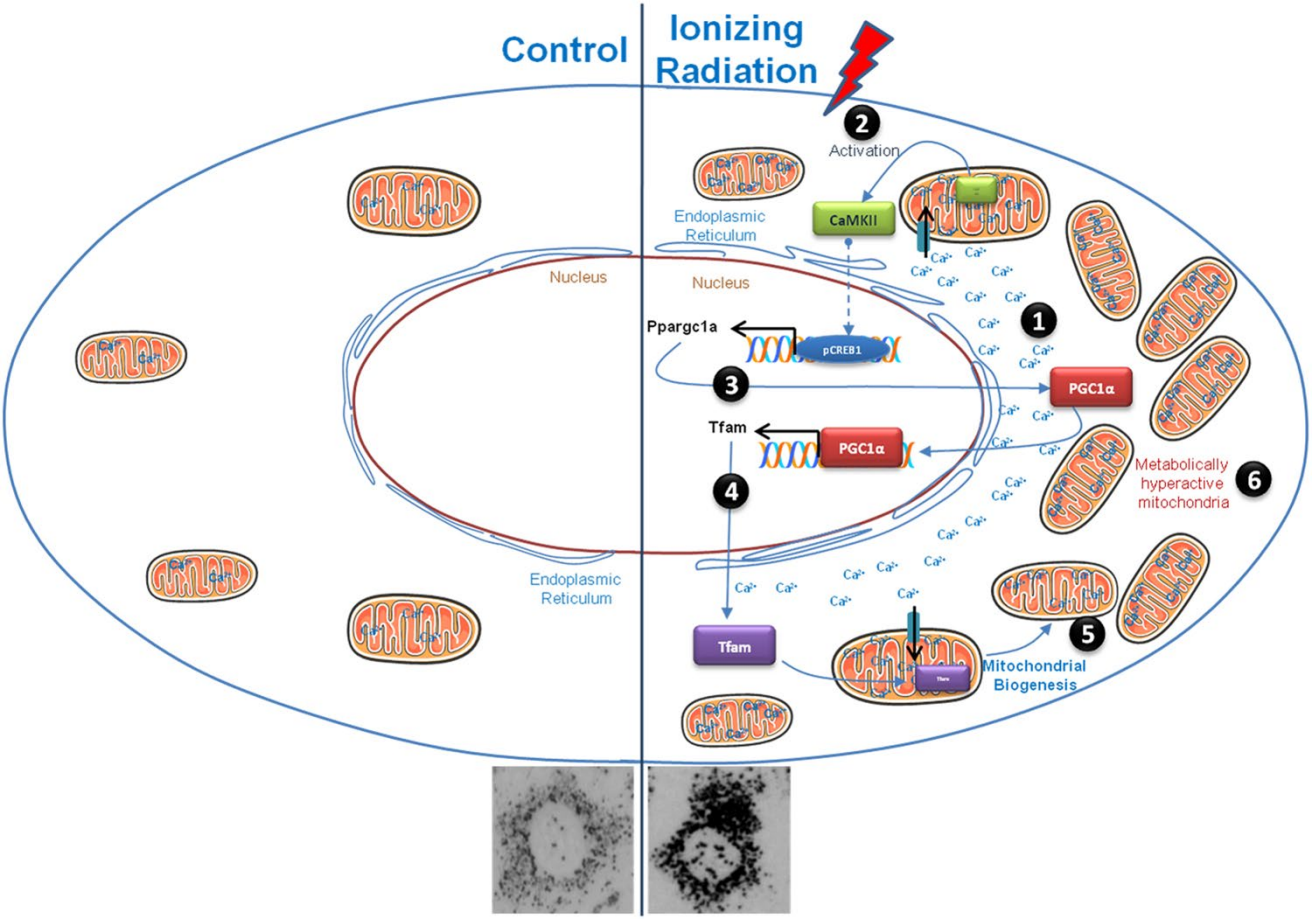
The diagram illustrates how radiation enhanced mitochondrial biogenesis and metabolic hyperactivation results in the increased formazan formation per cell. Ionizing radiation leads to increased intracellular Ca^2+^ (step 1), which further accumulates in mitochondria and activate mitochondrial biogenesis signaling (step 2). Activation of Ca^2+^ dependent signaling results in PGC-1α over-expression (step 3), which further transactivates the over-expression of Tfam (step 4), which induces mitochondrial biogenesis (step 5). Enhanced mitochondrial mass buffers cytoplasmic Ca^2+^ and turns in to metabolically hyperactive mitochondria (step 6).

These results support the hypothesis that radiation induced enhanced metabolic viability per cell is obtained by activating the mitochondrial biogenesis and accumulating the elevated cytoplasmic Ca^2+^ in increased mitochondrial mass. Elevated mitochondrial Ca^2+^ further increases the activity of mitochondrial dehydrogenases, which enhances the metabolic viability in radiation exposed cells (Fig. 7).

## Materials and Methods

### Materials

Dulbecco’s Minimum Essential Medium (DMEM), Penicillin G, streptomycin, nystatin, dimethyl sulfoxide (DMSO), 3-4,5-dimethylthiazol-2-yl)-2,5-diphenyltetrazolium bromide (MTT), Ruthenium Red, and BAPTA-AM were purchased from Sigma Chemicals Co. (St Louis, USA), where as Chloroamphenicol was obtained from Amresco USA. MitoSOX, TMRM, Fluo-3-AM, DiOC6, Mitotracker Red and Rhod-2 AM were procured from Molecular Probes (Eugene, USA). Primers were purchased from GCC biotech (India) while PCR master mix and hot start DNA polymerase was procured from Sigma, USA. Bhabhatron-II, a teletherapy machine from Panacea, Medical Technologies Pvt Ltd (Bangalore, India) with a dose rate of 1.24 Gy/min. was used as a source for γ ray (Co-60) irradiation.

### Sources of cell line

The mouse embryonic fibroblast (NIH/3T3), human embryonic kidney cells (HEK-293), human lung epithelial adenocarcinoma cells (A549), human embryonic mouse normal monocyte macrophage (Raw 264.7), mammalian breast carcinoma (MCF-7), its P53 mutant (MDA-MB-231), and cervix carcinoma (HeLa) were obtained from NCCS, Pune, India and cultured in their respective medium. Stock culture was maintained in the exponential growth phase by passaging them every 3 days with their respective growth medium supplemented with 10% foetal bovine serum and antibiotics in 60 mm tissue culture petri dish (BD Falcon, USA).

### Radiation treatment

All the experiments were carried out in 24 well plate, 60 mm and 35 mm tissue culture dishes. Exponentially growing cells were treated with either γ-radiation or drugs following overnight incubation at 37 °C in 5% CO_2_ in the incubator. The dose response analysis (2 Gy, 3 Gy, 5 Gy and 7 Gy) of γ-radiation was carried out in A549, HeLa and MDA-MB-231 cells with Co-60 gamma-rays. Further all experiments were carried out at single radiation dose of 5 Gy. The treatment schedule and concentration of other drug/inhibitors are mention in respective figure legends.

### MTT assay, Formazan quantification, cell counting and Formazan imaging

The cells were plated in 24 well culture plates (25000 cells/500 µl/well for all the cell lines except Raw264.7, which was seeded with 40,000 cells/well density) and incubated in CO_2_ incubator. Next day, treatment was given according to the experimental requirement. Further, at respective time points, 50 µl MTT solutions from the Stock (5 mg/ml) was added and cells were incubated in CO_2_ incubator in the dark for 2 hrs. The medium was removed and Formazan crystals formed by the cells were dissolved using 500 µl of DMSO followed by transfer in 96 well plate. The absorbance was read at 570 nm using 630 nm as reference wavelength on a Multiwell plate reader (Biotech Instruments, USA). Reduced formazan quantification was done with Formazan standard. At each respective time points cell numbers were counted with a Neubauer-improved counting chamber (Paul Marienfeld GmbH & Co. KG, Germany) under 10X objective, and 10X eye piece magnification with compound light microscope (Olympus CH30, Japan). After MTT incubation Formazan images were captured under bright field (20X objective) with inverted microscope (Olympus, Japan). All the parameters were recorded under similar experimental conditions.

### Measurement of mitochondrial mass, membrane potential, reactive oxygen species, intracellular and mitochondrial calcium

Quantitative analysis of mitochondrial content, ROS generation, intracellular and mitochondrial calcium in HeLa and MDA-MB-231 were carried out using Mitotracker Green (at respective time points, post irradiation), MitoSox, Fluo-3 AM, and Rhod-2 AM respectively. Cells were incubated with mitotracker green (100 nM; 15 min; 37 °C), MitoSox (5 µM; 20 min; 37 °C), Fluo-3-AM (5 µM; 30 min; 37 °C), DiOC6 (40 nM; 30 min; 37 °C) and Rhod-2 AM (1 µM; 20 min; 37 °C) in PBS (phosphate buffered saline), then washed with PBS and resuspended in PBS before analysis. The signals were recorded using LSR-II flow cytometer (BD, Mountainview, CA, USA).

### Imaging of mitochondrial membrane potential, intracellular and mitochondrial calcium

The treatment induced mitochondrial morphological changes and alteration in membrane potential was analyzed using Mitotracker red and TMRM. Intracellular and mitochondrial calcium imaging was carried out using Fluo-3 AM and Rhod-2 AM respectively. Cells were grown on sterile cover glass for microscopic examination. At respective time points, following incubation of TMRM (5 nM/ml; 30 min; 37 °C), Mitotracker Red (50 nM; 30 min; 37 °C), Fluo-3-AM (5 µM; 30 min; 37 °C), and Rhod-2 AM (1 µM; 20 min; 37 °C) in incubation buffer containing Magnesium chloride and Calcium chloride (except Fluo-3 AM) 1 mM each in PBS, cells were washed with PBS before examination. Images were captured on fluorescence microscope (Olympus BX 60 fluorescence microscope, Japan).

### Mitochondrial DNA content analysis

Mitochondrial DNA content was analyzed by semi-quantitative PCR. HeLa and MDA-MB-231 cells were plated in 60 mm petri dish with the density of 0.3 × 10^6^ and incubated overnight before irradiation (5 Gy). At respective time points cells were terminated and DNA was isolated using QIAamp® DNA Mini Kit. PCR primer sequences for amplification of mitochondrial DNA encoded tRNA-Leu gene and the nuclear encoded polγ gene (as positive control) are as follows:

tRNA-Leu gene (139 bp), Forward 5′-GATGGCAGAGCCCGGTAATCGC-3′

Reverse 5′-TAAGCATTAGGAATGCCATTGCG-3′.

For nuclear polγ gene (39 bp), Forward 5′-AGCGACGGGCAGCGGCGGCGGCA-3′

Reverse 5′-CCCTCCGAGGATAGCACTTGCGGC-3′

Final reaction volume of PCR reaction was 50 μl according to manufacturer protocol.

### Immuno-blot

The protein level of PGC-1α, TFAM, SDH-A and loading control β-Actin were determined in control and irradiated cells (HeLa and MDA-MB-231) by immunoblot analysis. Cells were cultured in PD60 incubated in CO_2_ incubator before treatment. Further cells were harvested post irradiation at various time points (8 hrs, and 24 hrs) and lysed in ice cold RIPA lysis buffer (Tris–HCl: 50 mM, pH 7.4, NP-40: 1%, NaCl: 150 mM, EDTA: 1 mM, PMSF: 2 mM, protease inhibitor cocktail, Na3VO4: 1 mM, NaF: 1 mM) containing protease inhibitors. The protein concentration in cell lysates was determined using BCA protein assay kit. Protein (60 μg) was resolved on 10–15% SDS–PAGE (depending on the molecular weight) and electroblotted onto PVDF membrane (MDI). The membrane was then incubated in 4% milk (according to manufacture protocol) for 2 h followed by primary antibody incubation SDH-A (1:1000), PGC-1α (1:1000), TFAM (1:1000) from Cell signaling technology, β-Actin (1:5000) from Santa Cruz Biotechnology. Membrane was washed followed by incubation with the appropriate HRP conjugated secondary antibody for 2 h. After washing, the blots were developed using ECL chemiluminescence detection reagent (Biological industries, Israel). The signal was detected by ECL and band intensities for each individual protein were quantified by densitometry, corrected for background staining, and normalized to the signal for β-Actin.

### Cell cycle analysis

The DNA content of cells in different phases of cell cycle was analyzed on flow cytometer. Exponentially growing cells were treated with radiation, further at respective time points (24 and 48 hrs), cells were harvested and fixed in 70% ethanol, then stored at −20 °C overnight. Further, cells were washed twice with cold PBS, resuspended in 200 µl PBS containing RNase A (200 µg/ml; 30 min; 37 °C) followed by propidium iodide staining (200 µl of PI 50 µg/mL; 15 min; 37 °C) and acquisition by flow cytometry. Data was analyzed on FlowJo software of BD, USA.

### Statistical analysis

All the experiments were carried out in triplicates or quadruplicates (mentioned in respective figure legends). Means and standard errors were computed. Student’s t test was quantified to determine the statistical significance between the groups.

## Electronic supplementary material

Supplementry information
