# Supplementary material for: Mitochondrial biogenesis and metabolic hyperactivation limits the application of MTT assay in the estimation of radiation induced growth inhibition
Source: Sci Rep. 2018 Jan 24;8:1531. doi: 10.1038/s41598-018-19930-w (PMC5784148; doi:10.1038/s41598-018-19930-w)
Supplement: Supplementary file 1 — Supplementry information [file 41598_2018_19930_MOESM1_ESM.pdf]

Mitochondrial biogenesis and metabolic hyperactivation limits the application of MTT assay in the estimation of radiation induced growth inhibition

Yogesh Rai[1], Richa Pathak[1], Neeraj Kumari[1], Dhananjay Kumar Sah[1], Sanjay Pandey[1], Namita Kalra[1], Ravi Soni[1], B S Dwarakanath[2] and Anant Narayan Bhatt[1]

Correspondence:
Anant Narayan Bhatt
Institute of Nuclear Medicine and Allied Sciences
Brig S K Mazumdar Road
Delhi 110 054
INDIA
Telephone: (+91) 11 2390-5138
Fax: (+91) 11 2391-9509
E-mail: anant@inmas.drdo.in, anbhatt@yahoo.com

Supplementary figure 1

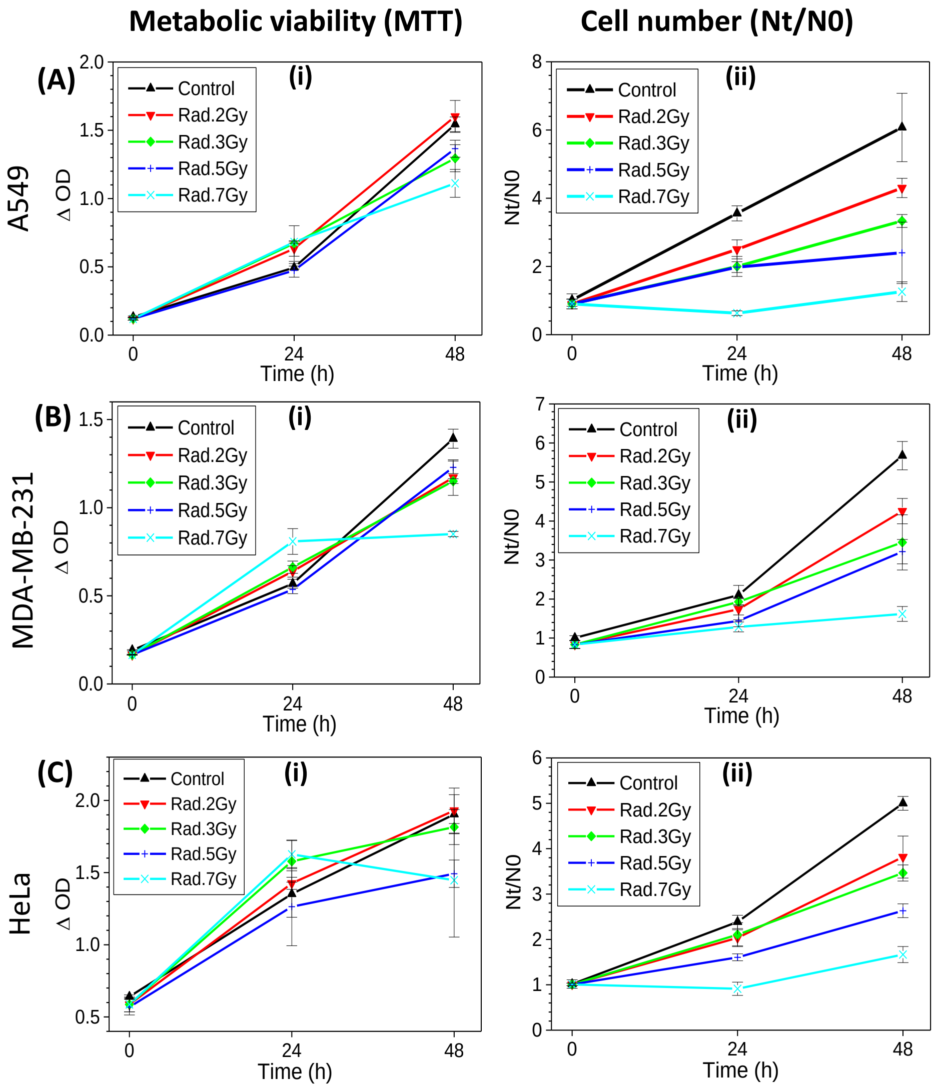

Radiation dose dependent analysis of metabolic viability (MTT) and cell number: Metabolic viability does not correlate with viable cell number in radiation induced growth inhibition: Radiation dose response measured by MTT assay in A549 (A i), MDA-MB-231(B i) and HeLa (C i) cells and compared with cell number in same cell lines A549 (A ii), MDAMB-231(B ii) and HeLa (C ii), under similar experimental conditions at various radiation dose. Data are presented as mean ± SD from four independent experiments. *p < 0.05 vs. respective control cells.

Supplementary figure 2

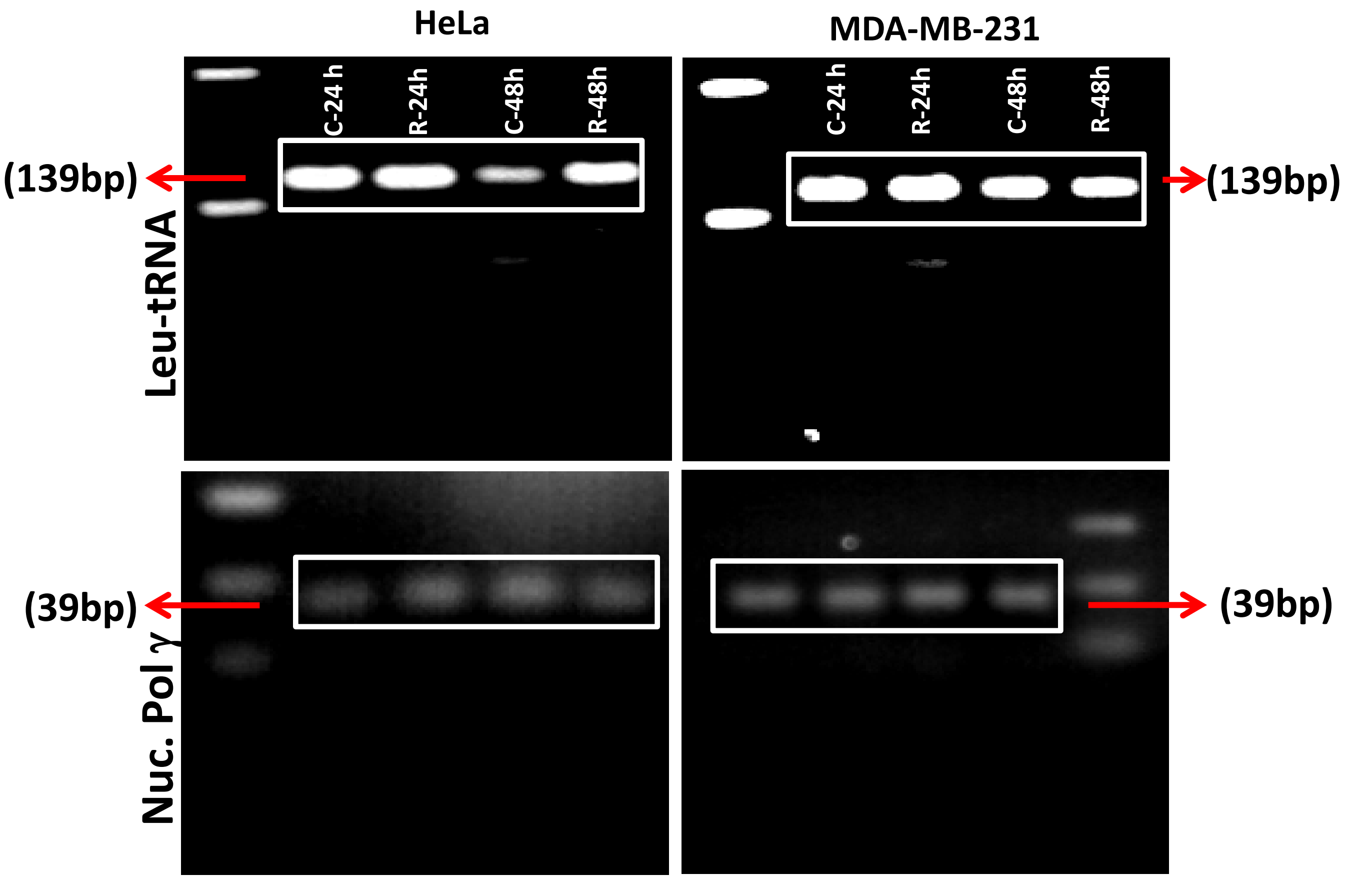

Figure 3 (B) is cropped and taken from these original images of the supplementary figure 1: Full length DNA gel blot images are presented here for Leu-tRNA (139 bp) and Nuclear pol γ (39 bp) in HeLa and MDAMB-231 cells at 24 and 48 hours post irradiation with respective control.

Supplementary figure 3

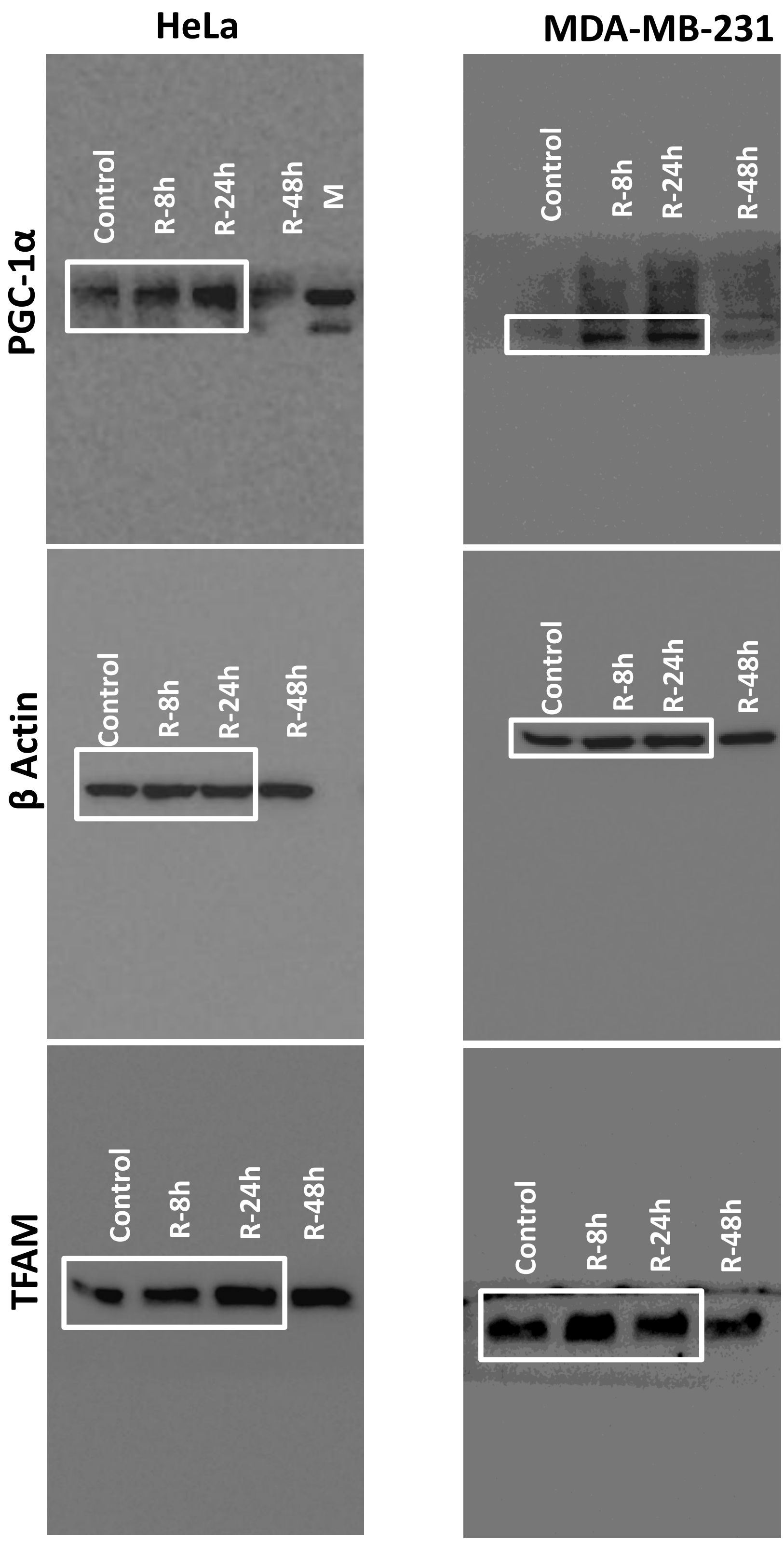

Supplementary figure 3 continue:

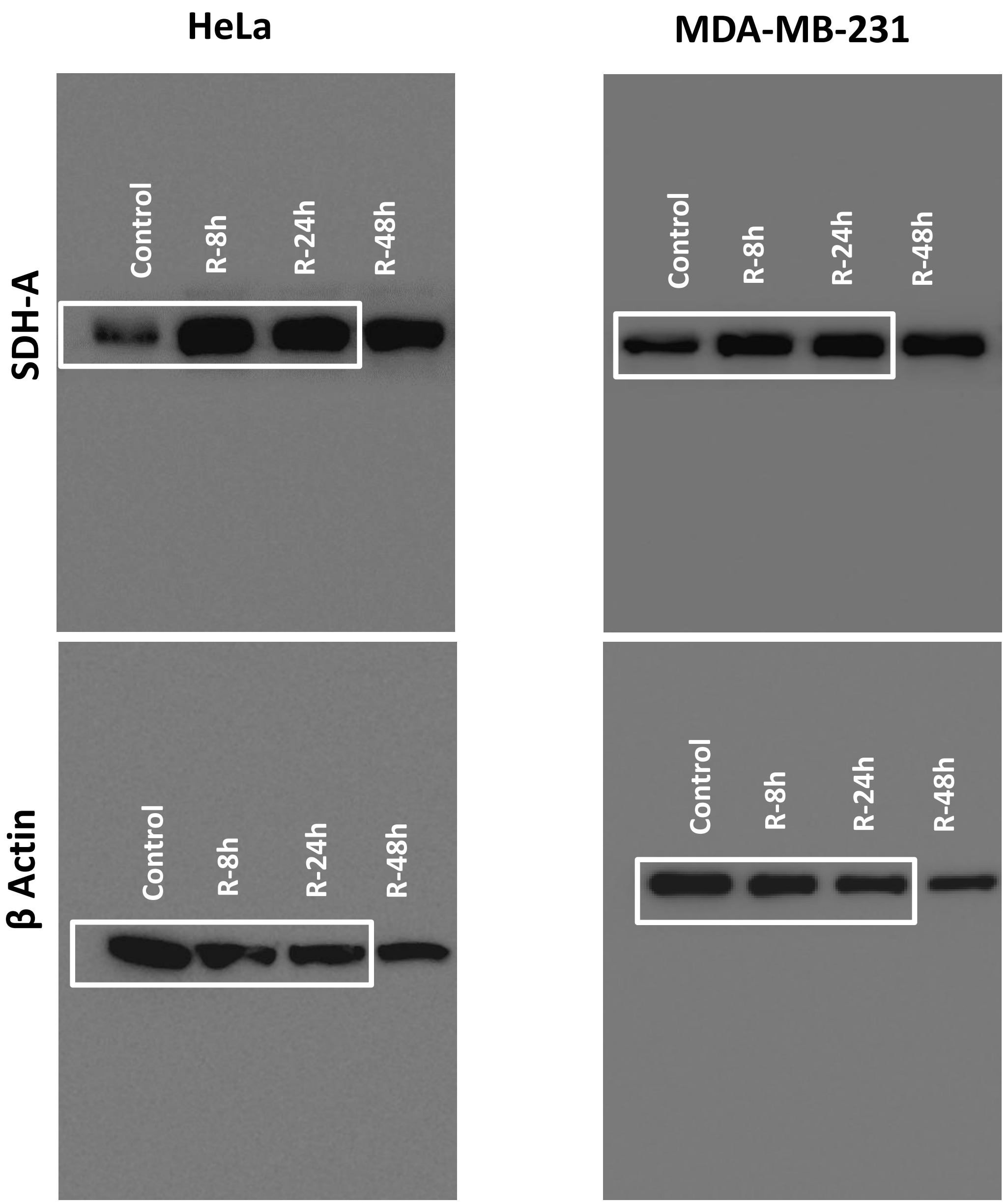

Figure 3 (C) is cropped and taken from these original images of the supplementary figure 3: Full length western blot images are presented here for PGC-1α, TFAM, SDH and its respective β Actin in HeLa and MDAMB-231 cells. Cropped images marked at 8, 24 and 48 hours with respective control and analyzed by densitometry.
